# A Comparative Analysis of the Morphology and Evolution of Permanent Sperm Depletion in Spiders

**DOI:** 10.1371/journal.pone.0016014

**Published:** 2011-01-11

**Authors:** Peter Michalik, Clare C. Rittschof

**Affiliations:** 1 Zoologisches Institut und Museum, Ernst-Moritz-Arndt-Universität, Greifswald, Germany; 2 Division of Invertebrate Zoology, American Museum of Natural History, New York, New York, United States of America; 3 Department of Biology, University of Florida, Gainesville, Florida, United States of America; University of Wyoming, United States of America

## Abstract

Once thought to be energetically cheap and easy to produce, empirical work has shown that sperm is a costly and limited resource for males. In some spider species, there is behavioral evidence that sperm are permanently depleted after a single mating. This extreme degree of mating investment appears to co-occur with other reproductive strategies common to spiders, e.g. genital mutilation and sexual cannibalism. Here we corroborate that sperm depletion in the golden orb-web spider *Nephila clavipes* is permanent by uncovering its mechanistic basis using light and electron microscopy. In addition, we use a phylogeny-based statistical analysis to test the evolutionary relationships between permanent sperm depletion (PSD) and other reproductive strategies in spiders. Male testes do not produce sperm during adulthood, which is unusual in spiders. Instead, spermatogenesis is nearly synchronous and ends before the maturation molt. Testis size decreases as males approach their maturation molt and reaches its lowest point after sperm is transferred into the male copulatory organs (pedipalps). As a consequence, the amount of sperm available to males for mating is limited to the sperm contained in the pedipalps, and once it is used, males lose their ability to fertilize eggs. Our data suggest that PSD has evolved independently at least three times within web-building spiders and is significantly correlated with the evolution of other mating strategies that limit males to monogamy, including genital mutilation and sexual cannibalism. We conclude that PSD may be an energy-saving adaptation in species where males are limited to monogamy. This could be particularly important in web-building spiders where extreme sexual size dimorphism results in large, sedentary females and small, searching males who rarely feed as adults and are vulnerable to starvation. Future work will explore possible energetic benefits and the evolutionary lability of PSD relative to other mate-limiting reproductive behaviors.

## Introduction

Sperm are small and numerous compared to eggs, which has lead to the assumption that sperm are cheap to produce. However, recent studies have shown that sperm production is costly, and males can become sperm depleted (i.e. functionally sterile) at least for some period of time after mating [1], [2], [3]. The amount of sperm transferred during copulation affects a male's fitness in terms of sperm competition for the current mate [4] and a male's ability to mate with subsequent females [5]. As a result, sperm cost and male sperm depletion have broad implications for the evolution of male mating strategies in sexually reproductive animals [6], [7], [8], [9].

Sperm depletion, the decrease in sperm number over successive ejaculates, is widespread in animals [10], but there is variation across species in the degree to which sperm depletion limits male mating opportunities [11]. Sperm depletion can be temporary, where males must undergo a reproductive latency period after mating in order to replenish their ejaculate (e.g. [12]). In other cases, sperm depletion is permanent, where males are unable to replenish their sperm once it is used [13]. Because permanent sperm depletion (hereafter PSD) strongly constrains male mating ability, the mechanistic, ecological, and evolutionary bases of this phenomenon are of special importance to a variety of research areas, including the evolution of mating systems, male mate choice, sperm competition, and female sperm limitation.

Web-building spiders are popular for male mating strategy studies because in some species males show a variety of behavioral and morphological features that limit mating rate, including male sacrifice behavior (i.e. sexual cannibalism, e.g. [14], [15], [16]) and complete or partial genital breakage during copulation (termed genital mutilation, e.g. [17], [18], [19]). Mating behaviors that eliminate a male's ability to re-mate are collectively called terminal investment strategies [20]. Because these behaviors occur across a variety of distantly related species [21], [22], many studies have examined the selection pressures that maintain these extreme behaviors, and whether these selection pressures can be generalized across spider taxa [23], [24], [25], [26], [27], [28].

However, some species of spiders exhibiting terminal investment strategies also show evidence of PSD. In order to interpret the evolutionary history, causes, and consequences of terminal investment strategies in spiders, it is necessary to verify the amount of sperm available to a male across successive copulations [29], [30]. Despite these broad implications, and although PSD was first suggested in spiders over 20 years ago [31], little is known about this phenomenon. Verifying whether PSD occurs, understanding its mechanistic basis, and assessing its evolutionary history are essential to interpreting the selection pressures driving terminal investment behaviors in this arthropod group.

The male reproductive system in spiders is unusual because males transfer sperm to the female using paired prosomal appendages called pedipalps that are separate from the male genital opening. After the maturation molt, males ejaculate sperm through their genital pore onto a sperm web and draw the sperm into their pedipalps, a process called sperm induction (see [32]). The sperm remains in the pedipalps until copulation. Studies that propose sperm depletion in spiders have examined the pedipalps for the presence or absence of sperm [31], which would indicate temporary sperm depletion because males can re-induct sperm before, during, or after copulation (Table 1). However, examination of the testes is required in order to demonstrate that sperm depletion is permanent. The male testes are simple, paired cylindrical organs that are connected to the genital pore by paired deferent ducts (see [33]). Sperm is produced in the testes and then temporarily stored in the deferent ducts until ejaculation. Cursory examination of the adult male testes suggests that sperm depletion may result because male testes atrophy or do not produce sperm during adulthood ([29], [30], Table 1).

**Table 1:** Overview of sperm depletion in spiders as it relates to sexual cannibalism and genital damage during copulation.

Family	Species	PSD	TSD	Sexual cannibalism	Genital damage	Source
**Nephilidae**	*Nephila clavipes*	yes	yes^F^	no	no	present study; [31]
	*Nephila plumipes*	?	no	yes	yes, partial genital breakage	[18], [23], [69]
	*Nephilengys malabariensis*	yes	?	yes	yes, emasculation	[58]; Michalik and Kuntner (unpublished)
**Tetragnathidae**	*Glenognatha emertoni*	?	no	no	no	[70]
	*Tetragnatha versicolor*	?	no	no	no	[71]
**Araneidae**	*Argiope keyserlingi*	yes	yes	yes	yes, partial genital breakage	[22], [29]
	*Argiope bruennichi*	?	yes^T^	yes	yes	[72], [73]
	*Micrathena gracilis*	?	yes^T^	no	no	[74]
	*Gasteracantha cancriformis*	?	yes^T^	14.7%**	no	[75]
**Theridiidae**	*Latrodectus hasselti*	no	yes	yes	yes	[76], [77]; Michalik and Andrade (unpublished)
	*Netiscodes rufipes*	?	yes^T^	no	no	[40], [78]
	*Tidarren argo*	yes	?	yes	yes, emasculation	[30], [41]
**Lycosidae**	*Schizocosa malitiosa*	no*	no	no	no	[79]
**Anyphaenidae**	*Anyphaena accentuata*	?	no	no	no	[80] (cited in [81])

In order to summarize direct evidence for sperm depletion in spiders, only studies which explicitly addressed sperm usage by sperm counts were considered. *PSD* (permanent sperm depletion; yes/no)*:* In cases where the testes were examined, was PSD confirmed? *TSD* (temporary sperm depletion; yes/no): In cases where the pedipalps were assessed for the presence of sperm after copulation, were the pedipalps sperm absent? The superscript T denotes that the occurrence of TSD depends on time spent copulating, and F denotes that the occurrence of TSD depends on the status of the female mate (i.e. virginity). Note that TSD does not necessarily suggest PSD since spider males usually re-induct sperm.

*males that were able to re-induct fathered significantly more offspring than those that were prevented from re-inducting.

**male is killed by female but not consumed.

In the current study, we verify that sperm depletion in spiders can be permanent using the golden orb-web spider *Nephila clavipes.* Spiders in the genus *Nephila* are known for genital mutilation and male sacrifice behavior[34], which suggests permanent sperm depletion may be present in this group. More importantly, extensive mating experiments in *N. clavipes* provide the best evidence of any spider species studied that sperm depletion may be permanent [31]. Using morphological measurements from specimens sacrificed at different life stages, we examine how the size of the testes changes as males mature, induct sperm, and age. We use light and transmission electron microscopy to confirm that decreased testes size corresponds to a decrease in the amount of tissue devoted to spermatogenesis. In addition, using a comparative phylogenetic framework and phylogeny-based statistics, we test the evolutionary relationships between PSD and other characters that limit male mating rate, including genital mutilation, male sacrifice behavior, and sexual size dimorphism.

## Results

In order to account for the effect of body size on testis size, we analyzed the variation in male body size and the relationship between body size and testis size. For males used in our analyses, body size showed limited variation; prosoma width ranged from 1.43 to 2.08 mm (Mean = 1.85 mm, SE±0.023 mm), and regression analyses showed that prosoma width was not significantly correlated with either testis or deferent duct volume (F_1,35_ = 0.022, R^2^ = 0.0006, P = 0.88; F_1,35_ = 2.55, R^2^ = 0.068, P = 0.12). For these reasons, we did not account for body size variation in any analyses of the genital system.

### Mechanistic basis of permanent sperm depletion

**Figure 1:**
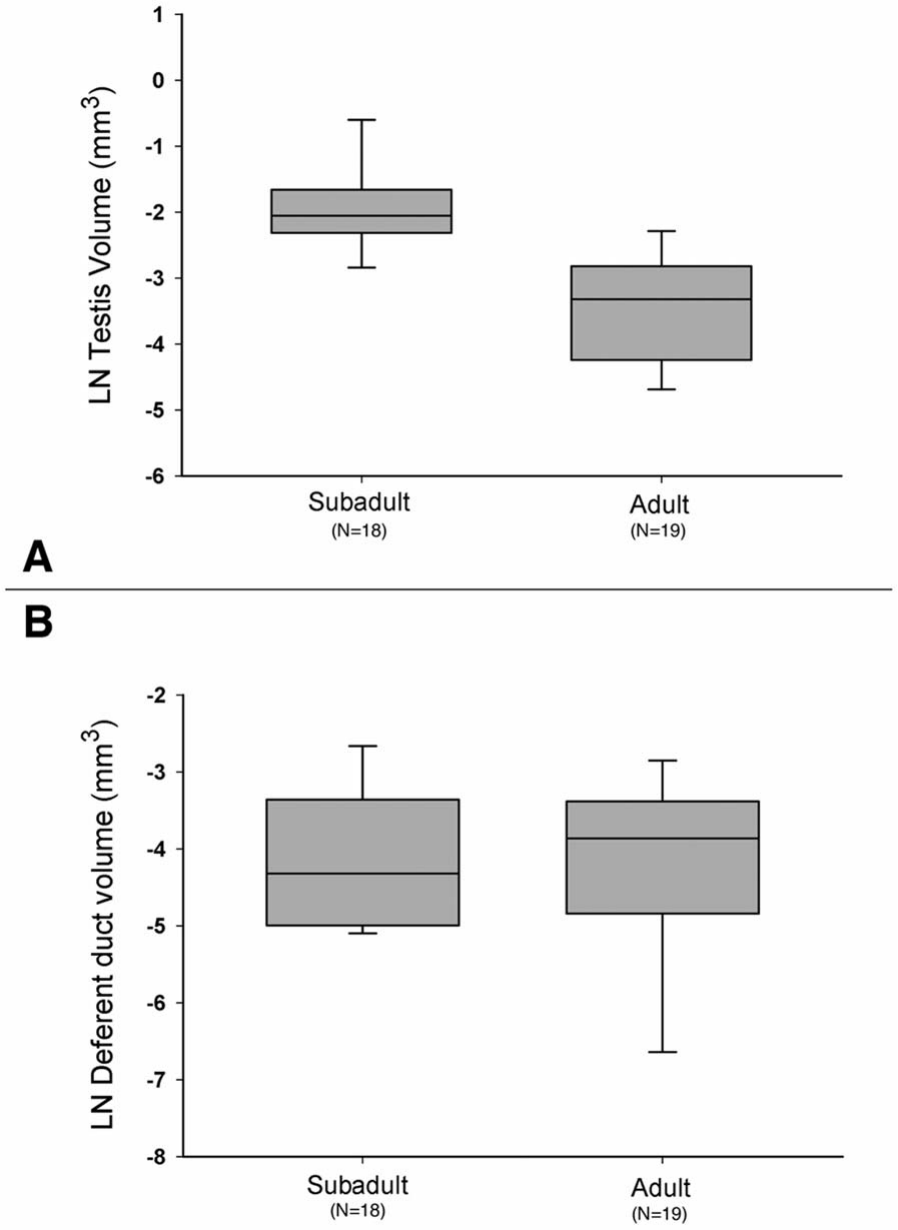
Testis volume vs. Deferent duct volume in *Nephila clavipes*. (A) Testis volume in sub-adult versus adult males (N*_subadult_* = 18, N*_adult_* = 19; F_1,35_ = 34.7; P<0.0001). (B) Deferent duct volume in sub-adult versus adult males (N*_subadult_* = 18, N*_adult_* = 19; F_1,35_ = 0.11; P = 0.73).

Testis volume was significantly greater for sub-adult males compared to adult males (two-tailed t-test, t_35_ = 5.89; P<0.0001; Fig. 1A). In contrast to testis volume, male deferent duct volume did not differ between sub-adult and adult males (two-tailed t-test, t_35_ = 0.34; P = 0.74, Fig. 1B). Thus, for our analyses, we focused on processes in the testis.

**Figure 2:**
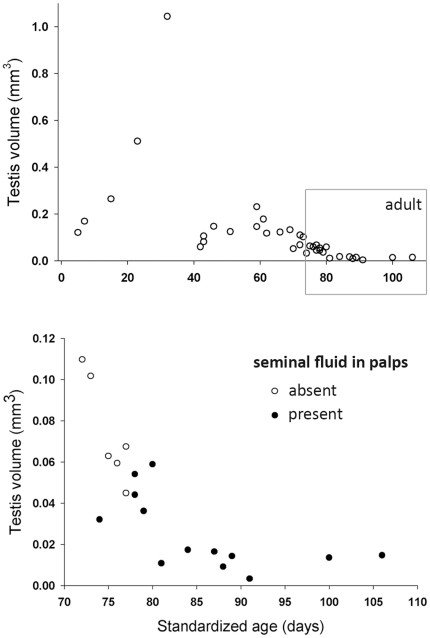
Trajectory of testis volume during male development in *Nephila clavipes*. The adult stage (box in upper graph) is enlarged to show the decrease of testis volume after sperm induction (Slope = −0.018, R^2^ = 0.72).

Male testis volume changed dramatically with male standardized age (Fig. 2A). Within the adult male group, testis volume showed a steep decline with male age that can be described as a negative logarithmic function (Fig. 2; Slope = −0.018, R^2^ = 0.72). The further decrease in volume for adult males is related to the induction of the seminal fluid into the male pedipalps (Fig. 2B).

**Figure 3:**
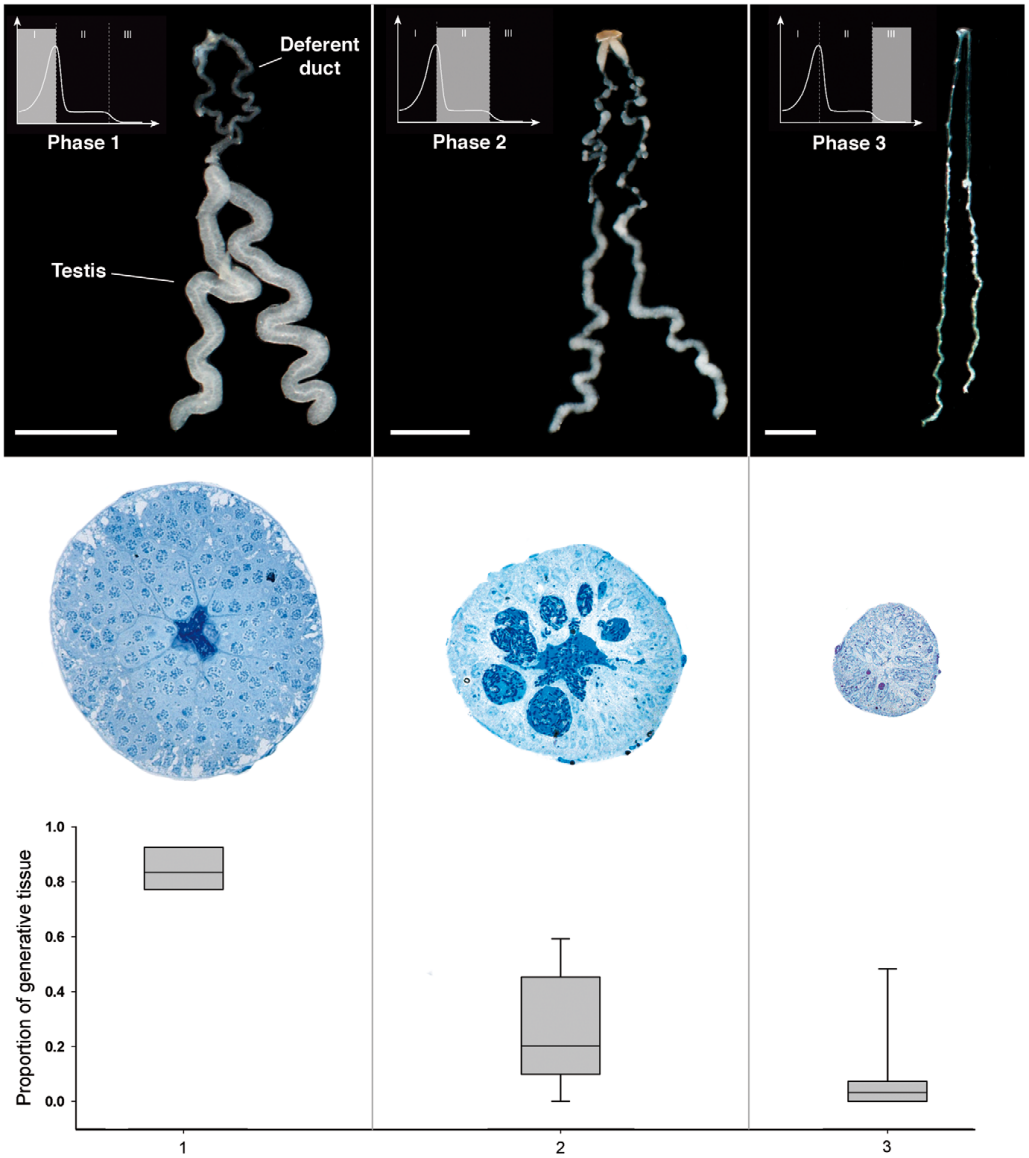
Development of the male genital system in *Nephila clavipes*. Three phases in the development of the male genital system (left to right) characterized by a dorsal view of the whole genital system (top, scale: 1 mm), a stained cross-section of the testis (middle), and the analysis of the proportion of the generative tissue in testis (bottom; Kruskal-Wallis Test, one-way; X^2^ = 17.26, P<0.0002).

**Figure 4:**
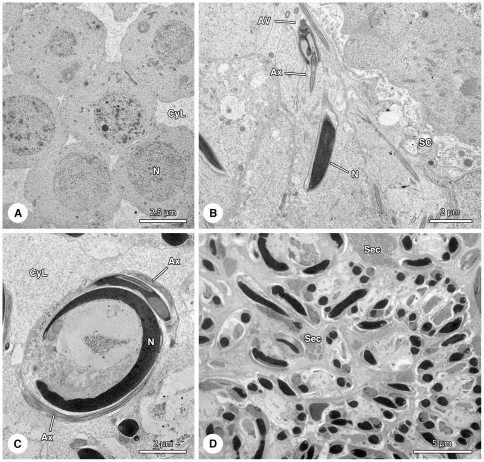
Ultrastructural aspects of spermatogenesis in *Nephila clavipes*. (A) Early spermatids. (B) Longitudinal section of mid-stage spermatids with condensed and elongated nuclei. (C) Coiled spermatid in a spermatogenic cyst. (D) Coiled sperm cells and secretion in the lumen of the testis. Abbreviations: AV, acrosomal vacuole; AX, axoneme; CyL, lumen of spermatic cyst; N, nucleus; SC, somatic cell; Sec, secretion.

The changes in testis volume corresponded to changes in the proportion of testis tissue devoted to sperm production. Combining all data, these changes over time can be described as three distinct phases (Fig. 3; Kruskal-Wallis Test, X^2^ = 17.26, P<0.0002). In phase one (Fig. 3, left), testis volume increases, and the majority of the testis tissue is devoted to sperm production. The peak testis volume corresponds to early spermatogenesis during which germ cells divide and spermatids start to differentiate nearly synchronously. The sperm cells in this phase are large and spherical (Fig. 4A). Over the course of spermatogenesis, the spermatids become more compact, indicated by the condensed chromatin in the nucleus and the relatively small amount of cytoplasm in the cell (Fig. 4B). At the end of spermatogenesis, the main cellular components coil within the sperm cell (Fig. 4C), a process that occurs in all other spiders studied (see [35]). The loss of volume of the spermatids due to the condensing and coiling processes induces phase two (Fig. 3, middle), where the spermatogenic cysts around the sperm cells contract, resulting in shrinking of the testes and a decrease in generative tissue. By the end of phase two, sperm cells have moved from the contracted cysts into the lumen of the testes (Fig. 4D) and they subsequently move into the lumen of the deferent duct. Sperm induction initiates phase three (Fig. 3, right); seminal fluid is discharged out of the testes and deferent duct through the genital opening. The absence of seminal fluid in the lumen results in further shrinking of the testes and near absence of generative tissue. The seminal fluid consists of spermatozoa and secretion (Fig. 4D), which is produced by the somatic cells of the testis.

### Evolutionary history of permanent sperm depletion

**Figure 5:**
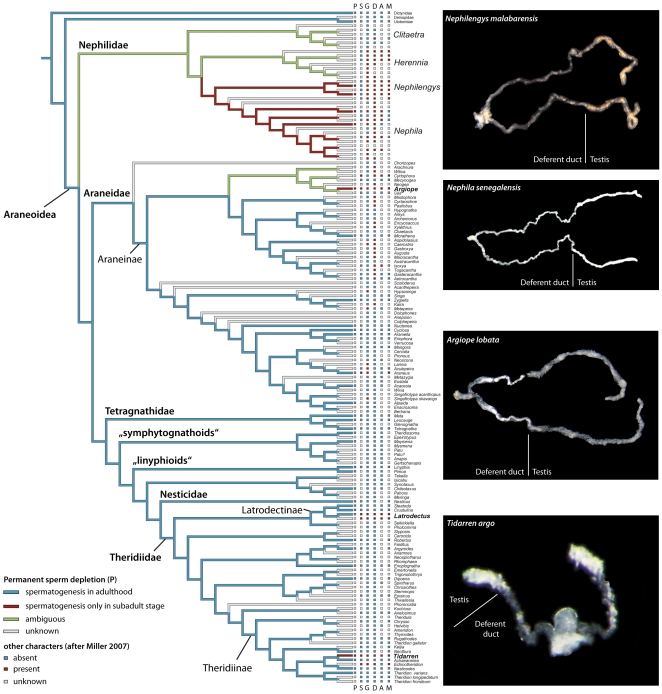
Evolution of PSD in araneoid spiders. PSD is optimized on the phylogeny of Araneoidea according to Miller [21] (topology “Kuntner et al. ((TA)(EN))”, Fig. 1 and supplemental material). Note the ambiguous optimization in the Nephilidae node and within Araneidae. Boxes indicate states of following characters: permanent sperm depletion (**P**), male sacrifice behavior (**S**), male genitalia mutilated in the course of copulation (**G**), extreme sexual size dimorphism (**D**), male accumulation (**A**), and male monogamy (**M**). The pictures in the right column show examples from all genera where PSD is present indicated by the equal diameter of deferent ducts and testes in adult male reproductive system (*Tidarren argo* taken from [30]).

The optimization of PSD suggests at least three independent origins of this trait in araneoid spiders, i.e. within Nephilidae, Araneidae and Theridiidae (Fig. 5). Based on the lack of behavioral data (scored as missing data, see also [21]), the presence of PSD in *Clitaetra* and *Herennia* (Nephilidae) as well as in the closest related taxa *Argiope* (Araneidae) is ambiguously optimized and should be addressed in future studies. As indicated by the CCT, the evolution of PSD is significantly correlated to and dependent on genital mutilation, male sacrifice, male monogamy, male accumulation, and sexual size dimorphism (Table 2).

**Table 2:** Hypotheses of the evolution of PSD tested using the concentrated change test for one of the phylogenies used by Miller ([21], topology "Kuntner et al. ((TA)(EN))").

independent	dependent	p-value	g, l of dep. (g ind.)
PSD	genital mutilation	0,52	+10, −4 (+3)
PSD	male sacrifice	0,49	+6 (+2)
PSD	male monogamy	0,086	+7, −1 (+3)
PSD	male accumulation	0,166	+6, −1 (+3)
PSD	size dimorphism	0,25	+7, −5 (+3)
genital mutilation	PSD	0,006	+3 (+3)
male sacrifice	PSD	0,0018	+3 (+2)
male monogamy	PSD	0,00069	+3 (+3)
male accumulation	PSD	0,005	+3 (+3)
size dimorphism	PSD	0,03	+3 (+3)

When optimization was ambiguous, accelerated transformations were preferred to minimize number of gains in the character state of interest. The right column indicates the total number of gains (g) and losses (l) of the dependent character (dep). The number of gains in the presence of the independent character (ind) is given in parentheses.

## Discussion

*Nephila clavipes* sperm maturation occurs only during the sub-adult instar, and spermatogenesis is completely absent in adult males. In addition, after the maturation molt, all sperm is inducted into the pedipalps at one time [36]. As a consequence, the amount of sperm available to males for mating is limited to the sperm contained in the male pedipalps, and once it is used, males lose their ability to fertilize eggs. These data strongly suggest that when males deplete their sperm, it is permanent.

PSD, characterized by nearly synchronous spermatogenesis and termination of spermatogenesis in the sub-adult instar, is unusual in spiders. In most spiders, spermatogenesis is ongoing throughout adulthood (e.g. [37]) and all stages of spermatogenesis are observed in the testes at the same time (e.g. [38]). Moreover, in most species, males generally recharge their pedipalps before, after or even during courtship and copulation (e.g. [39], [40]), indicating active sperm production in adult males.

PSD occurs in distantly related spider groups (Fig. 5). It has been documented in two spider families, Theridiidae (*Tidarren argo*; [30]), and Nephilidae (*Nephila clavipes* and *N. senegalensis; Nephilengys malabarensis* and *N. borbonica*; this study and Michalik and Kuntner unpublished data; Fig. 5). In addition, it has been suggested to occur in a third family, Araneidae (e.g. *Argiope keyserlingi*; [29]; see also Table 1 and below). Because these three families of spiders are distantly related, and because PSD occurs in some but not all members of these families, (Fig. 5; [30], Michalik, unpublished data), it is likely that PSD has evolved independently at least three times (Fig. 5). One plausible hypothesis for this distinct distribution of PSD is that the trait evolved in correlation with other traits that limit male reproductive rate [21].

Terminal investment behaviors, which eliminate male re-mating ability (e.g. male sacrifice behavior and genital mutilation [20]), often co-occur [21], and PSD appears to have evolved in lineages following the evolution of traits associated with male monogamy (Fig. 5, Table 2). Data suggest that in lineages with high levels of male competition (i.e. male accumulation; Fig. 5; [21]), which is often a consequence of extreme sexual size dimorphism [21], males are limited to monogamy, and genital mutilation and male sacrifice behaviors have evolved as mechanisms of paternity assurance (Fig. 5; [21], [22]). As a consequence, unlimited sperm production became unnecessary in these groups because males typically mate once (e.g. [15], [41]). The evolution of PSD may be an energy-saving mechanism that has evolved in some groups with terminal investment strategies and monogamy because it betters males' chances of locating and successfully achieving copulations with a female.

Sperm production and maintaining the function of the testes is energetically costly [42] particularly if males do not feed as adults. In the moth *Plodia interpunctella*, starved males show decreased sperm numbers [43]. Similarly, in the adder *Vipera berus*, males lose as much mass during periods of sperm production (when males are immobile) as they do during periods of active mate search [44]. In spiders, extreme sexual size dimorphism, which is a consequence of fecundity selection on females [45], [46], [ but see also 47], results in large sedentary females and mobile searching males. In species that require webs for prey-capture, males that search for females cannot build webs and so typically do not eat during adulthood (e.g. [48]), which makes them vulnerable to starvation.

The hypothesis that PSD is an energy-saving adaptation has some support in the genus *Tidarren* (Theridiidae). Males in this genus have unusually large pedipalps for their body size and so they castrate one of their pedipalps prior to sperm induction [41], [49]. Pedipalp removal increases male locomotor performance, giving males more stamina during mate search, and allowing males to find females more quickly [50]. The high locomotor performance that is characteristic of *Tidarren* is unusual among spider species [50]. Thus in this genus one result of increased energetic demands could be that males divert oxygen and other resources away from the testes and into the muscles leading to a loss of testes function (PSD; [30]).

The need for physical endurance is one characteristic that *Tidarren* appears to share with another sperm-depleting group, Nephilidae. Most of the Nephilid spiders have high post-copulatory energy requirements. Male sacrifice behavior is rare but genital mutilation is common, limiting male re-mating ability (Fig. 5; [21], [34]). However, males guard females after mating, which decreases the probability that the female will re-mate [48], [51], [52]. The termination of spermatogenesis might be an energy-saving measure that allows these males, who no longer have functional pedipalps, to spend extended periods of time fighting after copulation (up to two weeks in *N. clavipes*; [48]).

In contrast, in the genus *Latrodectus* (Theridiidae), the widows, male sacrifice behavior is common, but preliminary results suggest that PSD does not occur in this genus (Michalik et al. unpublished data for *L. hasselti*, *L. hesperus* and *L. geometricus*; Table 1). However, in *Latrodectus*, even when males are not cannibalized during copulation, they die soon after mating [53]. Thus, *Latrodectus* species do not appear to share the level of physical stamina and survivorship found in *Tidarren* and the Nephilids, possibly because *Latrodectus* species do not have the same pre and post-copulatory energetic demands. This preliminary hypothesis might explain the conspicuous absence of PSD in *Latrodectus*, even though these species are well known for high rates of genital mutilation, male sacrifice behavior, and monogamy [25], [54]. Future studies should address this problem.

In the third family of spiders that show PSD, the Araneids, PSD is only found in *Argiope* ([29], Michalik et al. unpublished; Fig. 5), one of the few genera in this family that has high rates of genital mutilation (e.g. [55], [56]) and male sacrifice behavior (e.g. [15], [16]). Although extreme sexual size dimorphism is common in the Araneids, it does not typically lead to monogamy in this group [21], which may explain the rarity of PSD in the family. Thus although sexual size dimorphism can lead to monogamy in some cases [21], [57], the patterns in the Araneids suggest that either genital mutilation or male sacrifice behavior, which impose the strongest constraints on male re-mating opportunity, must be present for PSD to evolve. Further analyses of the Araneids should test for PSD more broadly to determine whether it occurs only in groups with genital mutilation or sacrifice behavior, or if extreme sexual size dimorphism alone (which only somewhat constrains male mating rate) is sufficient to favor its evolution.

It is important to note that there is variation in the occurrence of genital mutilation within the family Nephilidae, even though PSD may be present in all species (see Fig. 5). In *Nephila fenestrata*, males commonly break off the distal tip of the embolus during mating rendering the pedipalp useless [27], while in other species (e.g. *N. edulis*), embolus breakage is rare or absent [22], [34]. In *N. clavipes* specifically, the evidence for embolus breakage is equivocal [22], [34], and recent work in *Nephilengys borbonica* has shown that genital mutilation is a labile trait that occurs only in certain mating contexts [58].

In the Nephilids, character optimization and CCT suggests that genital mutilation preceded the evolution of PSD, and mutilation was secondarily lost in certain species ([34], Fig. 5, Table 2). The loss of genital mutilation could have occurred because changes in the species' mating systems allowed males to benefit from mating multiply, or because females escaped male plugging behavior over evolutionary time (i.e. antagonistic co-evolution of genitalia; [34]). However, while males in these species may have regained the ability to maintain intact pedipalps during copulation, they may not be able to regain functional testes once PSD has evolved. Instead, in some species (e.g. *N. clavipes*) males may prudently allocate sperm, particularly when mating with non-virgin females ([59]; Rittschof in press). Thus it remains unclear whether PSD results in monogamy in all cases. Future studies should confirm the occurrence of PSD in Nephilids, determine the relationship between PSD and male mating rate, and examine the evolutionary lability of PSD relative to other terminal investment behaviors. The Nephilid spiders, which show a broad range of mating systems and terminal investment behaviors, make an ideal group for this comparative study.

### Conclusion

Here we verify that sperm depletion in *Nephila clavipes* is permanent, and describe its mechanistic basis. Cursory studies in other species suggest that spiders exhibiting PSD share this common mechanism [30]. Although PSD is an unusual phenomenon, it appears to have evolved multiple times in association with genital mutilation, male sacrifice behavior, and other traits associated with monogamy (e.g. sexual size dimorphism). In general, PSD could be an energy-saving adaptation, although the factors favoring it (e.g. pre-copulatory mate search or post-copulatory mate guarding) may be species-specific. Future work will explore the costs of sperm production, the energetic benefits of PSD, and employ a broad comparative phylogenetic approach to address the relationships between PSD, male terminal investment behaviors, and environmental factors that constrain male mating rate in spiders.

## Materials and Methods

### Collection and Rearing

Third and fourth instar juvenile *Nephila clavipes* were collected from mixed oak habitats within the Ordway-Swisher Biological Station in Melrose, Florida (Putnam County). Juveniles were reared at the Lab of General and Systematic Zoology of the University of Greifswald in cylindrical plastic containers (5×10 cm). The tops of the containers were covered with cheesecloth, and the bottoms were open. The open bottoms sat on top of moistened towels to provide humidity, and males were housed together on a shelf under natural light cycle at room temperature. In the containers males constructed prey-capture webs and were fed daily with two *Drosophila* flies.

Because we were interested in comparing changes in the testes as males approach and pass their maturation molt, we sacrificed sub-adult males across a range of times that spans the period between the sub-adult and adult molt (see Table S1: Real age and standardized age for all males in the study). Furthermore, we sacrificed adult males across a range of times that spans the period from the maturation molt to death (N_sub-adult_ = 18, N_adult_ = 19; see Table S1). Our experimental set-up simplifies the true reproductive experience for adult males because we did not allow males to mate, nor did we provide females as cues for sperm production. However, in a series of behavioral experiments in this species, Christenson [31] gave males the opportunity to copulate with multiple receptive virgin females in succession, and males failed to transfer any sperm after their first mating in this scenario [31]. This suggests that any processes occurring in the male genital system that limit re-mating ability are independent of male mating history and female cues.

### Sample dissection and preparation

Virgin male specimens were dissected in phosphate buffer (0.1 M, pH 7.2) with 1.8% sucrose added (PB). The isolated genital systems were fixed in 2.5% glutaraldehyde (Merck Chemicals Ltd., Nottingham, UK) in PB and pictures for the analyses of the gross morphology were taken using an Olympus DP10 digital camera mounted on an Olympus ZX 7 stereomicroscope. For the histological and transmission electron microscope (TEM) analyses samples were post-fixed in PB buffered 2% OsO_4_ (SERVA Electrophoresis GmbH, Heidelberg, Germany). After being washed in PB, the genital system was dehydrated in graded ethanol and embedded in Spurr's resin [60]. For the light microscope (LM) analyses semi-thin sections (700 nm) were made with a Diatome HistoJumbo diamond knife at a Leica ultramicrotome UCT and stained according to Richardson et al. [61]. Sections were documented using a Zeiss MCr digital camera mounted on an Olympus BX60 compound microscope. For the TEM analyses ultra-thin sections (50 nm) were made with a Diatome Ultra 35° diamond knife at a Leica ultramicrotome UCT and stained with uranyl acetate and lead citrate according to Reynolds [62]. Examination was performed with a JEOL JEM-1011 electron microscope at 80 KV. Images were taken with a side-mounted Olympus MegaView III digital camera using the iTEM software (Olympus Soft Imaging Solutions GmbH, Münster, Germany).

### Measurements and Calculations

In order to compare changes in the genital system before and after the maturation molt, it was necessary to evaluate both male age groups along a single continuum of age. For this reason, adult males were assigned a “standardized age” that is the age at which they were sacrificed relative to the sub-adult males in the study (Table S1: Real age and standardized age for all males in the study). For example, the oldest sub-adult male was sacrificed on day 72 after his sub-adult molt, so the adult sacrificed one day after his own maturation molt received an age of 73.

In order to determine how the male reproductive system changes with age, measurements of body size, testis and deferent duct width and length, and the ratio of generative tissue to total testis tissue were taken for all males in the study (N = 37). All body measurements were taken from digital photographs (Zeiss Discovery V20 with Zeiss MCr camera) using the IntMess module in the program Zeiss AxioVision 4.8 (Carl Zeiss MicroImaging GmbH, Göttingen, Germany). A linear measurement of male body size was taken as the width of the prosoma at its widest point. In addition, after dissection, the length and width of one testis and one deferent duct were measured per individual. For each individual, the most intact of each organ was selected for measurement. Because of their irregular shapes, total testis and deferent duct lengths were measured using the curve tool of the IntMess module, which traces along the length of non-linear objects. Widths of the testis and deferent duct were taken as linear measurements. Because testis and deferent duct widths vary along the length of both organs, the width of each organ was measured at three locations per individual, the 25%, 50%, and 75% points along the length. These widths were averaged to estimate the true organ width. Using the assumption that the testis and deferent duct are approximately cylindrical, the length and average width measurements were used to calculate testis and deferent duct volumes.

To assess changes in generative tissue over male lifetime, we calculated the ratio of generative tissue to total testis area from a single stained testis cross-section per individual. Cross-sections were magnified using an Olympus BX60 light microscope and photographed with a Zeiss MCr camera. In spiders, spermatogenesis occurs in cysts, which are bordered by thin extensions of the somatic cells located at the periphery of the testis (e.g. [63]). At the end of spermatogenesis, sperm cells accumulate in the lumen of the testis. Thus, we defined generative tissue as the total area of the testis filled with either spermatogenic cysts or lumen. The borders of the testis, the testis lumen, and the spermatogenic cysts were traced using the IntMess module to approximate area. The resulting testis cysts area and lumen area were summed, and this value, divided by the total testis area, gives the ratio of generative tissue to total testis tissue.

### Male sperm induction

In order to assess whether the process of sperm induction corresponds to changes in the testes, we assessed male pedipalps for the presence of sperm. To do this, for each adult male in the study (N = 19), we removed the left pedipalp and soaked it in clove oil (Sigma Aldrich Chemie GmbH, Munich, Germany) for 3–5 hrs and then examined the pedipalp under a light microscope. Using this method, we could visualize sperm through the pedipalp cuticle in order to determine sperm presence or absence.

### Statistics

All statistical analyses were performed using the SAS program JMP 7.0 (SAS Institute Inc., Cary, NC, USA). For comparisons of testis and deferent duct volume for sub-adult versus adult males, the data were natural-log transformed in order to normalize the data distributions, and analyzed using two-tailed t-tests. All other data were analyzed without transformation. Male generative tissue changes were analyzed using a Kruskal-Wallis Test.

### Character optimization and phylogenetic comparative analyses

In order to assess the evolutionary history of PSD and other male mate-limiting characteristics in spiders, we elaborated on Miller's [21] phylogenetic hypotheses of araneoid spiders, which assessed the evolutionary history of genital mutilation, male sacrifice behavior, male accumulation on female webs (i.e. male-biased operational sex ratio), and monogamy in this group. All analyses were based on the trees and character matrix of Miller ([21]; see for detailed description of the used phylogenetic hypotheses and characters: [1] male genitalia typically mutilated in the course of copulation (**G**), [2] male sacrifice behavior (**S**), [3] extreme sexual size dimorphism (**D**), [4] male accumulation (**A**), and [5] male monogamy (**M**)). Additionally, we coded the character **“permanent sperm depletion”** (**P**) based on the present data and published and unpublished results ([30], [63], [64], [65]; Michalik, unpublished observations; Dataset S1) as follows: 0, *spermatogenesis ongoing in adulthood*; 1, *spermatogenesis only in subadult stage*. Although the phylogenetic hypothesis used by Miller [21] is based on morphological data it represents to date one of the most comprehensive and accepted phylogenetic hypothesis for Orbiculariae, particularly for resolving relationships among the Nephilids.

The character optimization was carried out with the Maximum Parsimony method (MP) implemented in the software Mesquite 2.7.2 [66]. To evaluate whether origins of one specific character state (“dependent character”) is more concentrated on branches with another specific character state (“independent character”) we performed the concentrated changes test (CCT; [67]). The CCT was run in MacClade 4.0 [68] using 100,000 replicate simulations with the ancestral state unspecified and actual changes considered. We analyzed the correlation between PSD and genital mutilation, male sacrifice behavior, sexual size dimorphism, monogamy, and male accumulation based on the four phylogenies used by Miller [21]. Since the tests in all four phylogenies resulted in nearly identical p-values we restricted Table 2 to the results based on the phylogenetic hypothesis shown in Fig. 5 (topology “Kuntner et al. ((TA)(EN))” [21]).

## Supporting Information

Table S1 Real age (number of days after penultimate or final molt when sacrificed) and standardized age (age relative to the youngest sub-adult male) for all males in the study. For following standardized age more than one specimen was used: 43, 59, 77, 78.
(PDF)

Dataset S1 Matrix and phylogenies used for the analyses.
(TXT)
