# Supplementary material for: A Comparative Analysis of the Morphology and Evolution of Permanent Sperm Depletion in Spiders
Source: PLoS One. 2011 Jan 11;6(1):e16014. doi: 10.1371/journal.pone.0016014 (PMC3019211; doi:10.1371/journal.pone.0016014)
Supplement: Table S1 — Real age (number of days after penultimate or final molt when sacrificed) and standardized age (age relative to the youngest sub-adult male) for all males in the study. For following standardized age more than one specimen was used: 43, 59, 77, 78. (PDF) [file pone.0016014.s001.pdf]

| Subadults | | | | | | | | | | | | | | | | |
|---|---|---|---|---|---|---|---|---|---|---|---|---|---|---|---|---|
| Age | 5 | 7 | 15 | 23 | 32 | 42 | 43 | 46 | 51 | 59 | 61 | 62 | 66 | 69 | 70 | 72 |
| Standardized age | 5 | 7 | 15 | 23 | 32 | 42 | 43 | 46 | 51 | 59 | 61 | 62 | 66 | 69 | 70 | 72 |

| Adults | | | | | | | | | | | | | | | | | |
|---|---|---|---|---|---|---|---|---|---|---|---|---|---|---|---|---|---|
| Age | 0,04 | 1 | 2 | 3 | 4 | 5 | 6 | 7 | 8 | 9 | 12 | 15 | 16 | 17 | 19 | 28 | 34 |
| Standardized age | 72.04 | 73 | 74 | 75 | 76 | 77 | 78 | 79 | 80 | 81 | 84 | 87 | 88 | 89 | 90 | 99 | 104 |
